# Transcriptomic Alteration in FUS-ALS Points Towards Apoptosis-Rather than Ferroptosis-Related Cell Death Pathway

**DOI:** 10.3390/cells14181417

**Published:** 2025-09-10

**Authors:** Banaja P. Dash, Andreas Hermann

**Affiliations:** 1 Translational Neurodegeneration Section “Albrecht Kossel”, Department of Neurology, University Medical Center Rostock, 18147 Rostock, Germany; banaja.dash@med.uni-rostock.de; 2 Center for Transdisciplinary Neurosciences Rostock, University Medical Center Rostock, 18147 Rostock, Germany; 3 German Center for Neurodegenerative Diseases (DZNE), Rostock/Greifswald, 18147 Rostock, Germany

**Keywords:** ferroptosis, apoptosis, ferroptosis-related genes, ALS, iPSC, motor neuron, differentially expressed genes, RNA-sequencing, gene expression omnibus

## Abstract

Amyotrophic lateral sclerosis (ALS) is a fatal type of neurodegenerative disease marked by progressive and selective degeneration of motor neurons (MNs) present in the spinal cord, brain stem and motor cortex. However, the intricate molecular mechanisms underlying primary cell death pathways, including ferroptosis-related genes (FRGs) mediating MN dysfunction in ALS, remain elusive. Ferroptosis, a novel type of iron-dependent cell death with the accumulation of lipid peroxidation products, stands distinct from apoptotic-related stress and other cell death mechanisms. Although growing advances have highlighted the role of iron deposition, apoptosis and alteration of antioxidant systems in ALS pathogenesis, there is little data at the systems biology level. Therefore, we performed a comprehensive bioinformatic analysis of bulk RNA-sequencing (RNA-seq) data by systematically comparing the gene expression profiles from iPSC-derived MNs of ALS patients and healthy controls using our datasets as well as from the GEO database to reveal the role of ferroptosis-related gene alterations in ALS, especially in selective MN vulnerability of *FUSED IN SARCOMA* (*FUS*) mutations. In this study, we first identified differentially expressed genes (DEGs) between *FUS* mutant and healthy controls. Subsequently, the crossover genes between DEGs and FRGs were selected as differentially expressed ferroptosis-related genes (DEFRGs). Functional enrichment and protein–protein interaction (PPI) analysis of DEFRGs identified that DNA damage, stress response and extra cellular matrix (ECM) were the most significantly dysregulated functions/pathways in *FUS*-ALS causing mutations compared to healthy controls. While GSEA analysis showed enrichment of genes associated with apoptosis, the degree of ferroptosis and iron ion homeostasis/response to iron of *FUS* MNs was lower. Altogether, our findings may contribute to a better understanding of the relevant role of cell death pathways underlying selective vulnerability of MNs to neurodegeneration in *FUS*-ALS pathophysiology.

## 1. Introduction

Amyotrophic lateral sclerosis (ALS) is a progressive and irreversible neurodegenerative disease characterized by the selective loss of motor neurons (MNs) in the motor cortex, brain stem and spinal cord, leading to progressive muscle weakness and death from respiratory failure typically within 2–5 years of symptom onset. While the majority of ALS cases are sporadic (sALS), the remaining 5–10% of cases are familial (fALS) with mutations found in over 40 Mendelian inherited genes [1]. Among those, mutations in *C9orf72*, *SOD1*, *FUS*, *TARDBP* and *TBK1* are the most prevalent in European populations and have the highest penetrance [2].These causative genes encode proteins with different functions, and the pathogenesis has been implicated with defects in stress response, mitochondrial dysfunction, hyperexcitability, impaired protein homeostasis, aberrant RNA metabolism, and impaired DNA repair [3,4]. In ALS, MN death is recognized to be both cell-autonomous, driven by intrinsic cellular defects or genetic mutations, and non-cell autonomous, caused by the external factors or detrimental signals from other cells (e.g., astrocytes, microglia) [2].

Despite extensive research studies having been performed, the etiology of ALS MN degeneration, including the primary cell death mechanisms responsible for MN death, remains poorly understood. This prevents the development of effective interventions and precise therapy stratification. Cell death is a vital biological process that contributes to development, homeostasis maintenance and disease prevention in multicellular organisms. Cell death is broadly classified into two groups: programmed/regulated cell death (energy-dependent) and non-programmed/necrotic cell death (energy-independent) [5,6]. Programmed cell death is defined by strictly regulated mechanisms and encompasses orchestrated signaling cascades operated at the molecular level and further divided into apoptotic or non-apoptotic programmed cell death. In contrast, non-programmed cell death represents an uncontrolled biological process and occurs under the influence of accidental cell damage [6,7]. MN death was initially implicated to be apoptotic, the most extensively studied form of cell death, and triggered by the intrinsic pathway (internal stress such as DNA damage, mitochondrial dysfunction leading to caspase activation), and the extrinsic pathway (activation of death receptors, tumor necrosis factor or p75 neurotrophin receptors) [8]. However, recent scientific investigations have reported an emerging repertoire of non-apoptotic modalities of MN cell death, leading to the implication of ferroptosis in the pathogenesis of various neurodegenerative diseases including ALS [9,10,11,12,13].

Ferroptosis, an iron- and lipid-peroxidation (LPO)-dependent form (and caspase-independent form) of non-apoptotic cell death [14], is typically induced by the excessive accumulation of iron and lipid reactive oxygen species (ROS) and/or inactivation of cellular antioxidant systems, contributing to neuronal damage. More recently, abnormality of iron homeostasis and anomalous accumulation of iron have been implicated in ALS patients (motor cortex, spinal cord and cerebral regions) [14,15,16,17,18] and animal models [19,20,21,22,23], respectively. Furthermore, a research study using induced pluripotent stem cell (iPSC)-derived MNs from sALS patients showed the involvement of lipid peroxidation and ferroptosis in MN cell death [24]. In addition, previous studies in ALS patients have demonstrated that cell death triggered by lipid peroxides (iron-dependent regulated necrosis) resulted in significant downregulation of endogenous mechanisms involved in protecting cells against ferroptosis, such as the glutathione peroxidase 4 anti-oxidant defense checkpoint (*GSH*/*GPX4*), and has been associated with degeneration of MNs and disease progression in ALS (sALS and fALS), suggesting a potential link between ALS and ferroptosis [11,16,25]. Consequently, the overexpression of *GPX4* in transgenic *SOD1* mice significantly mitigates symptoms and improved motor neuron function [23,26]. Additionally, recent evidence explored ferroptotic cell death in *FUS*-ALS, uncovering mitochondrial disturbances and heightened vulnerability to ferroptosis in *FUS* P525L MNs [18].

Although few research studies have been carried out on complex mechanisms of ALS and ferroptosis, their specific relationship on a more system biology level such as transcriptomics remains unclear. Since the *FUS* core function is to regulate transcription, we wanted to systematically investigate how much mutations in *FUS* affect ferroptosis-related gene (FRGs) expression. In the present study, using iPSC-derived MNs expressing *FUS*-ALS mutations, we performed a comprehensive bioinformatics analysis by systematically analyzing five RNA-sequencing (RNA-seq) datasets, including our dataset from the publicly available gene expression omnibus (GEO) database. We employed integrated differential expression gene (DEG) analysis to identify target genes and altered pathways related to ferroptosis in MNs between *FUS* mutant and healthy controls (WT), which contributes to further explore the pathomechanisms and selective MN vulnerability in *FUS*-ALS.

## 2. Materials and Methods

### 2.1. RNA-Seq Data Acquisition, Processing and Screening of DEGs

According to the main purpose of our study, publicly available RNA-seq datasets of “ALS patients versus healthy controls” using a search criteria including keywords and/or combinations relating to human, iPSC, MNs, ALS, bulk RNA-seq and *FUS* mutations were downloaded from the NCBI GEO database (https://www.ncbi.nlm.nih.gov/geo/, accessed on 10 September 2024). With this inclusion criteria, we could only find four RNA-seq datasets (studies 1 to 4) that could be used in combination with our study (study 5) for integrated analysis. A summary of the datasets used is shown in Table 1. FASTQ files of each study (see Table 1) were processed and analyzed using *Partek™ Flow™* software, v11.0 following the standard pipeline within the software to quantify gene counts as described in [27,28]. Briefly, FASTQ files were quality-checked (QA/QC), bases and reads with low quality were filtered out and adaptors were trimmed from the raw data. Reads were aligned to the reference human genome [hg38//GRCh38 (obtained from Ensembl assembly v100)] using the STAR v2.7.8a aligner [29]. Quality control analysis was performed on aligned reads to assess read quality using the QA/QC task. Processed aligned reads were then quantified against the Ensembl v100 hg38/GRCh38 human reference genome using the Expectation Maximization (EM) algorithm [30]. Gene counts obtained in each individual RNA-seq study were further combined to identify DEGs across datasets. Next, a noise reduction filter was applied to exclude genes considered as background noise, and genes that were not expressed by any cell in the dataset were filtered out. Finally, normalization and differential gene expression of the raw read counts were performed using DESeq2 (v1.16.1) at the gene level [31] with a statistically significant threshold of Benjamini–Hochberg false discovery rate (FDR) ≤ 0.05 and |log_2_FC| ≥ ±1. The normalized datasets were adjusted for batch effects using the General linear model method. The effectiveness of batch effect removal was confirmed by conducting principal component analysis (PCA) on the normalized counts of the dataset both before and after the batch correction was eliminated, and this aids to visualize similarities and differences between the samples in the dataset while identifying potential outliers. Statistically significant DEGs selected from the integrated dataset were clustered in a hierarchical manner using the correlation distance method and displayed in a heatmap. A volcano plot was used to visualize the distribution of DEGs. All above analyses were generated using *Partek™ Flow™* software, v11.0. Differential expression analysis results are provided in Supplementary Table S1.

### 2.2. Identification of DEGs Associated with Ferroptosis

We retrieved a well-documented list of human FRGs (322 genes) including drivers, suppressors or markers from the public Ferroptosis database (FerrDb V2; http://www.zhounan.org/ferrdb, accessed on 24 December 2024) [32]. In addition, we also used the GeneCards database (https://www.genecards.org, accessed on 24 December 2024) [33], which provides comprehensive information on human genes. The term “ferroptosis” was used as the keyword for the search to identify genes related to ferroptosis (732 genes), with a relevance score ≥ 1, and protein-coding-related genes. Next, the targets retrieved from the above two databases were merged, and the final list of FRGs was collected after removing duplicate genes. Next, we intersected the ferroptosis-related genes list with DEGs to identify common ferroptosis-related DEGs (DEFRGs) between the *FUS*-ALS patients and WT controls, and a total of 31 DEFRGs were screened out (as shown in the Supplementary Table S2) using the Venn diagram and selected for further analysis. The list of FRGs and DEFRGs is provided in Supplementary Table S3.

### 2.3. Functional Enrichment Analysis of DEGs and DEFRGs

To better understand the biological functions of DEGs and DEFRGs associated with *FUS*-ALS and the related signaling pathways involved, we performed a functional enrichment analysis using the database for annotation, visualization and integrated discovery, DAVID version 6.8 (https://david.ncifcrf.gov/summary.jsp, accessed on 30 April 2025, enrichment testing across all GO functions, pathways) online platform [34]. GO enrichment was applied to annotate and analyze genes involved in biological process (BP), molecular function (MF), and cellular component (CC) gene function annotations. Pathway enrichment analysis was carried out using Kyoto Encyclopedia of Genes and Genomes (KEGG) and Reactome databases. Functional analysis was carried out using default settings, and a *p*-value ≤ 0.05 was considered to be a statistically significant enrichment.

### 2.4. Protein–Protein Interaction Network Analysis

We generated a PPI network for DEFRGs using the Search Tool for the Retrieval of Interacting Genes/Proteins (STRING) database (https://string-db.org/) [35] with the minimum required interaction score ≥ 0.15. The nodes represent the DEGs/proteins, and the edges indicate the predicted functional interactions (databases, high-throughput experiments, co-expression/co-occurrence, text mining, neighborhood genes) between two proteins. Subsequently, the PPI network was imported into Cytoscape 3.10.3 software [36] to build a visual network and execute topological analysis. The key nodes were selected according to the scoring of maximal clique centrality (MCC) by using the cytoHubba [37], a plug-in for Cytoscape that explores important nodes and subnetworks by topological algorithms. Ten genes scoring the highest were identified as hub genes in our study. These hub genes may play essential roles in regulating ferroptosis and warrant further investigation.

### 2.5. Gene Set Enrichment Analysis (GSEA)

To study the functional alterations in pathways and biological processes of the samples in the expression datasets, we performed gene set enrichment analysis (GSEA) [38] on the normalized gene counts (DESeq2) of RNA-seq data (mutant and control) using the GSEA function in *Partek*^TM^ *Flow*^TM^ software, v11.0. The used gene sets for testing were the GO-term-derived gene set database (biological process, molecular function, and cellular component) and KEGG pathway database. The metric for ranking gene parameters was signal-to-noise, and the significant gene sets/pathway enrichment was identified by the normalized enrichment score (|NES| ≥ 1) and *p* ≤ 0.05.

### 2.6. Statistical Analysis

The general pipeline for combined RNA-seq analysis used in this study, including alignment, quantitation, normalization and differential gene expression analysis as well as statistical analyses were performed on *Partek*™ *Flow*™ software, v11.0 to aid the visualization and interpretation of the expression patterns of DEGs. When appropriate, *p*-values (*p*-value ≤ 0.05) were calculated and adjusted for multiple testing using FDR correction statistical methods for DEGs using *Partek*™ *Flow*™ software, v11.0. *p*-values/FDRs ≤ 0.05 and log_2_FC ≥ 1 or log_2_FC ≤ −1 were considered as statistically significant thresholds for the identification of DEGs. Unpaired, two-tailed Student’s t-tests were performed using GraphPad Prism v9.4.1. software to analyze gene expression levels.

## 3. Results

### 3.1. Data Processing and Identification of DEGs

**Figure 1:**
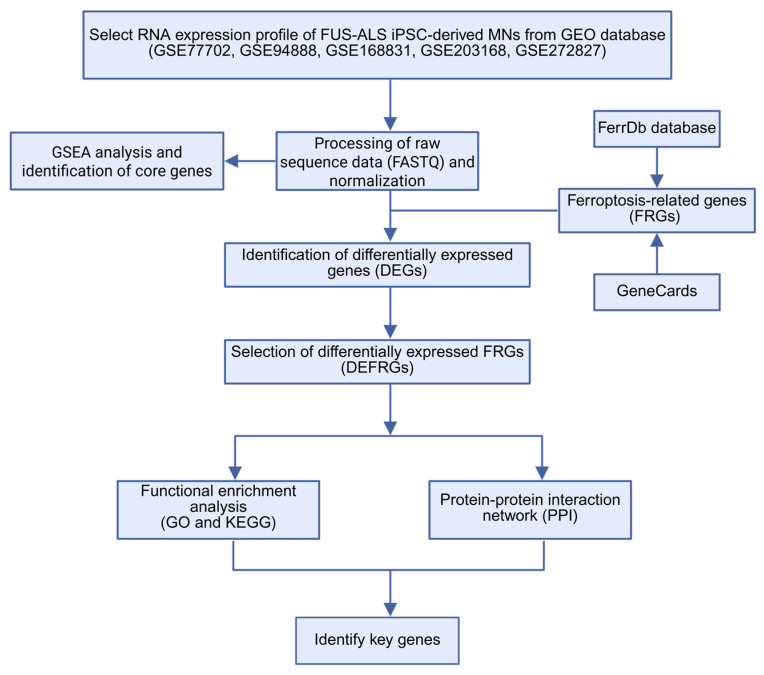
The workflow of this study. ALS, amyotrophic lateral sclerosis; DEG, differentially expressed genes; FRG, ferroptosis-related gene; DEFRGs, differentially expressed ferroptosis-related genes; GO, gene ontology; KEGG, Kyoto Encyclopedia of Genes and Genomes; PPI, protein–protein interaction.

**Figure 2:**
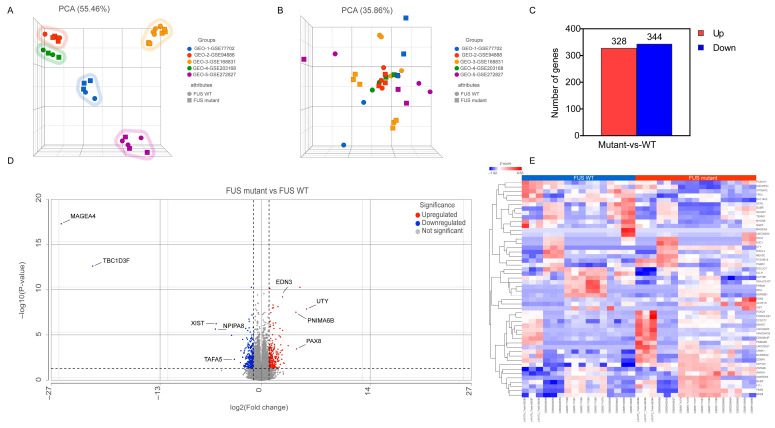
Transcriptomic data analysis. (**A**) Principal component analysis (PCA) of samples before batch effect removal and (**B**) after batch-effect removal, highlighting similarities and differences in the gene expression between the various *FUS* samples compared to WT samples. In the three-dimensional PCA plot, each sample is represented as a sphere/square; the closer the spheres/squares in the spreadsheet, the higher the similarity among sample groups. A total of 33 types of samples from 5 different datasets are shown on this figure; different colors indicate different datasets, while different symbols (sphere/square) represent sample attribute types (WT; sphere and *FUS* mutant; square). (**C**) Statistics on the results of differentially expressed genes (DEGs) in the *FUS* samples. (**D**) Volcano plot displaying the distribution of DEGs between *FUS* mutant and WT controls (*p* ≤ 0.05, FDR ≤ 0.05 and log_2_Fold Change) ≥ 1 or log_2_(Fold Change) ≤ −1). (**E**) Heatmap representing the expression profile of the 50 DEGs identified by *FUS* mutant versus WT controls (*p* ≤ 0.05, FDR ≤ 0.05, |log_2_FC | ≥ 1). Expression values sorted according to sample (rows) and gene (columns), where the color change from red to blue suggests gene expression changing from high to low.

We searched relevant RNA expression profiles from the GEO database and focused our analysis on iPSC-derived spinal MN studies from *FUS*-ALS patients and age-matched WT controls. As a result, five gene expression datasets, GSE77702 [39], GSE94888 [40], GSE168831 [41], GSE203168 [42] and GSE272827 [43], were collected and considered for further analysis (Table 1). The workflow of the study is shown in Figure 1. After background noise correction, all the datasets were pooled, followed by normalization. The PCA plot demonstrated a clear separation of individual *FUS*-ALS datasets from the remaining groups (Figure 2A). The batch effect across ALS samples was removed effectively using PCA, highlighting that no such separate clusters were observed (Figure 2B). The combined dataset obtained after processing and batch effect correction gave rise to an initial dataset consisting of 33 samples (17 ALS samples and 16 WT controls) and 36,828 genes, henceforth referred to as the final dataset. Next, differential expression analysis was performed between *FUS*-ALS and WT control samples to identify DEGs based on the threshold of FDR ≤ 0.05 and |log_2_FC (Fold Change) | ≥ 1. A total of 672 DEGs were identified after removing genes without the HGNC annotation ID, containing 328 upregulated and 344 downregulated genes, which were visualized using a volcano plot and heatmap (Figure 2C, displayed in Supplementary Table S1). The volcano plot showed the distribution of gene expression between *FUS*-ALS and control groups (Figure 2D), whereas the clustered heatmap of DEGs revealed the distinct patterns of upregulated or downregulated genes across the samples grouped by genotype (Figure 2E).

**Table 1:** Summary of five gene expression profiles derived from GEO database.

Study	GEO Dataset	Platform	Mutation	Gender (Age)	Sample Size (n) ALS/Control	Library Type	Layout	DIV	References
Study 1	GSE77702	GPL11154 Ilumina HiSeq 2000	*FUS* ^R521G^	N/A	3/2	PolyA	Single	34	[39]
Study 2	GSE94888	GPL16791 Illumina HiSeq 2500	*FUS* ^P525L^	Female (20) (*FUS*^wt/P525L^)	3/3	Ribo-zero	Paired	19	[40]
Study 3	GSE168831	GPL24676 Illumina NovaSeq 6000	*FUS*^R495QfsX527^ (c.1483delC), frameshift *FUS*^Asp502ThrfS*27^ (c.1504delG), frameshift	*FUS*^wt^: Female (45), Male (64), Male (46) *FUS*^R495QfsX527^ (c.1483delC), frameshift: Male (26) *FUS*^Asp502ThrfS*27^ (c.1504delG), frameshift: Male (19)	6/6	PolyA	Paired	35	[41]
Study 4	GSE203168	GPL20301 Illumina HiSeq 4000	*FUS* ^H517Q^	(mean ± SD, 4 Patients) 45.0 ± 3.6 (age: N/A)	2/2	Ribo-zero	Single	30	[42]
Study 5 (our study)	GSE272827	GPL18573 Illumina NextSeq 500	*FUS* ^P525L^	Isogenic control: *FUS*^WT^-eGFP^het^: Female (58) Isogenic mutant: *FUS*^P525L^-eGFP^het^ *FUS*^R521C het^ Female (58)	3/3	PolyA	Single	21	[43]

DIV: days in vitro.

### 3.2. Functional Enrichment Analysis of DEGs

**Figure 3:**
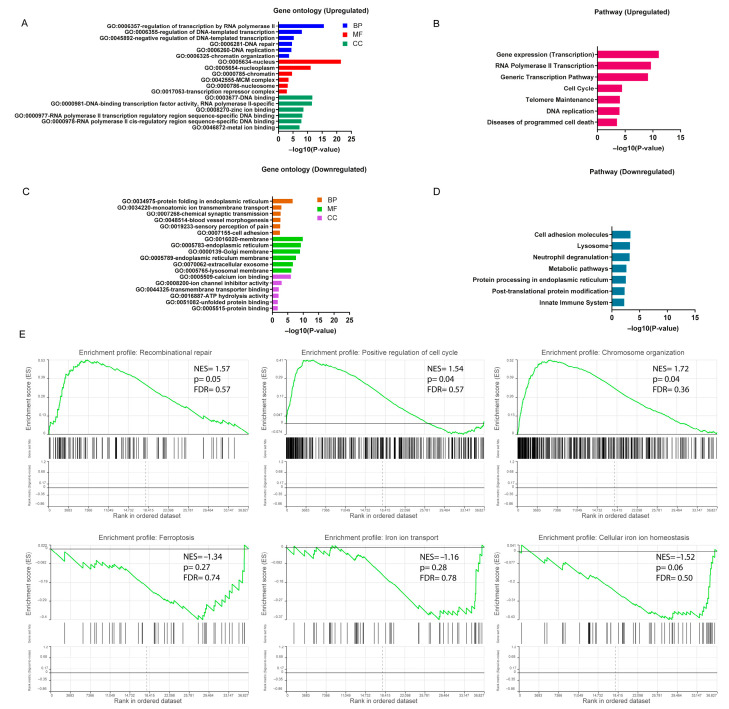
Functional enrichment analysis of DEGs. (**A**) Bar plot of gene ontology (GO) enrichment analysis of upregulated DEGs. (**B**) Pathway enrichment analysis of upregulated DEGs. (**C**) Bar plot of gene ontology (GO) enrichment analysis of downregulated DEGs. (**D**) Pathway enrichment analysis of downregulated DEGs. GO enrichment analysis was based on the topmost significant enriched terms in each of the biological process (BP), cellular component (CC) and molecular function (MF) entries (*p* ≤ 0.05). (**E**) Gene set enrichment analysis (GSEA) was performed using normalized counts from the DESeq2 output ranked list in *FUS*-ALS datasets (*p* ≤ 0.05 was considered statistically significant). The top three most significantly enriched gene sets were shown to be positively and negatively correlated with the gene counts based on NES value. The y-axis represents the enrichment score for the overall gene set, and on the x-axis are genes (vertical black bars) represented in gene sets.

To better understand the underlying potential biological functions, we performed GO and pathway (Reactome and KEGG) enrichment analysis on the 672 DEGs. The GO analysis results (*p*-value ≤ 0.05) showed that upregulated DEGs were significantly enriched in various biological processes, including regulation of transcription by RNA polymerase II and DNA repair and chromatin organization. DEGs were more likely to be involved in the nucleus, nucleoplasm and chromatin complex in the cellular components category. In terms of molecular functions, DEGs were mainly involved in DNA binding functions. The top six of the biological process, cellular component and molecular function enriched terms are shown in Figure 3A, Supplementary Table S2. Pathway enrichment analysis revealed significant enriched terms, including gene expression, DNA replication, RNA polymerase II transcription and cell cycle pathway (Figure 3B, Supplementary Table S2). Considering the MNs as the main focus of this study, the comprehensive enrichment analysis indicated crucial molecular alternation in DNA repair and relevant functions in *FUS*-ALS MNs.

For the downregulated DEGs, significant biological processes included protein folding in endoplasmic reticulum (ER), ion membrane transport and synaptic transmission. The cellular component enrichment analysis showed important terms, including organelle membrane, ER and extracellular exosome. Among the molecular functions, the most enriched functions were ion binding, ion channel activity and transporter binding. The significant GO-enriched terms are displayed in Figure 3C. The KEGG analysis indicated that DEGs participated in the cell adhesion pathway, lysosome and metabolic pathways (Figure 3D, Supplementary Table S2).

### 3.3. GSEA Analysis in FUS-ALS

To further explore the impact of gene expression levels on *FUS*-ALS phenotypes (*FUS* mutant and WT controls), we used the GSEA algorithm to investigate the relationship between gene expression in the combined dataset and the biological processes, cellular components, molecular functions and KEGG pathways involved (*p* ≤ 0.05) (Supplementary Table S3, Figure 3E, Supplementary Figures S1 and S2). The results demonstrated that genes in the combined dataset were significantly enriched in cellular functions such as DNA repair, cell cycle and chromosome organization pathways, and positively correlated with the expressed genes, while cellular metabolism, cell adhesion, iron ion homeostasis, iron ion transport, ubiquitination, synaptic/neuronal and organelle functions were negatively correlated with the expressed genes. In addition, the GSEA results also revealed that the ferroptosis pathway enrichment was inhibited in the *FUS* MNs (ferroptosis: NES = −1.34, *p* = 0.27, leading edge size = 14; *VDAC2*, *FTH1*, *SLC3A2*, *ATG7*, *MAP1LC3C*, *SLC7A11*, *SLC39A14*, *MAP1LC3B*, *ACSL1*, *GCLM*, P*RNP*, *ACSL4*, *TF* and *MAP1LC3B2*), whereas the apoptosis-enriched gene set, including the *p53*-signaling pathway, neuron apoptosis process, ROS levels and antioxidant activity, were mostly upregulated (apoptosis: NES = 0.74, *p* = 0.86, leading edge size = 29) (Figure 3E, Supplementary Figures S1 and S2 and Supplementary Table S3). However, most likely due to the small sample set size, the ferroptosis- and apoptosis-related gene set missed statistical significance in the enrichment analysis. Nevertheless, these results suggested that there was a significant difference between the *FUS* mutant and WT control groups at the transcriptional level, and the DNA repair as well as the cell death-related terms including ferroptosis/iron homeostasis pathways were markedly changed in *FUS*-ALS. We hypothesize that ferroptosis may play a certain role in the disease progression of *FUS*-ALS. Thus, to substantiate the role of FRGs differentially expressed in the *FUS* MNs, the DEGs obtained from GEO gene expression datasets were intersected with the ferroptosis gene set to identify DEFRGs. The detailed DEFRG results are presented below and in Supplementary Table S4.

### 3.4. Identification of Differentially Expressed Ferroptosis-Related Genes (DEFRGs)

**Figure 4:**
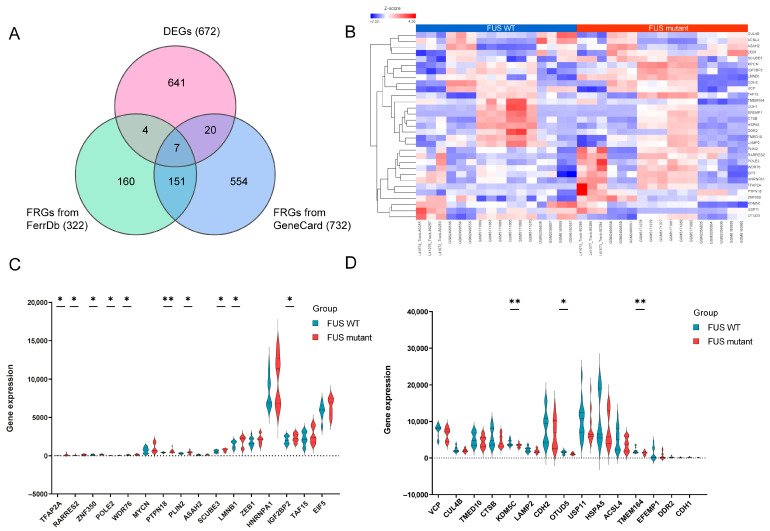
Identification of differentially expressed ferroptosis-related genes (DEFRGs). (**A**) Venn diagram identifying the 31 overlapping DEFRGs between DEGs in GEO datasets (672) and ferroptosis-related genes (322) in the FerrDB and GeneCard (732) databases, respectively. FRGs, ferroptosis-related genes; DEFRGs, differentially expressed ferroptosis-related genes. (**B**) Heatmap displaying the expression of 31 DEFRGs in *FUS* and healthy controls (WT). (**C**,**D**) Violin plots illustrating the gene expression levels of 31 DEFRGs between *FUS* mutant and WT controls. (**C**) A total of 16 upregulated genes (**D**) 15 downregulated genes, blue for WT group, and red for *FUS* mutant group; the X-axis shows the ferroptosis-related genes, and the Y-axis represents the expression level of genes. Student’s t test was employed to assess the gene expression values between the mutant and WT groups. The asterisks indicate that the differences are statistically significant (* *p* ≤ 0.05, ** *p* ≤ 0.01).

To deepen the analysis on a potential relationship between ferroptosis and *FUS*-ALS, 322 and 732 FRGs were collected from FerrDb and GeneCards databases, respectively. After removing duplicate genes, a total of 896 FRGs were obtained by combining the targets retrieved from the above two databases. Using a Venn plot, we intersected FRGs with the DEGs in *FUS*-ALS and identified 31 overlapped DEFRGs, including 16 upregulated and 15 downregulated genes for further analysis (Figure 4A). The expression levels of 31 DEFRGs in *FUS*-ALS are shown in the heatmap (Figure 4B). The violin plot shows the expression patterns of 31 significantly DEFRGs, which were expressed differentially between *FUS* mutant and WT control samples (Figure 4C,D). Details of overlapping genes (out of 31 DEFRGs) from the FerrDb, including six drivers and five suppressors, are presented in Table 2. All the FRGs and DEFRGs are listed in Supplementary Table S4.

### 3.5. Enrichment Analysis of DEFRGs

**Figure 5:**
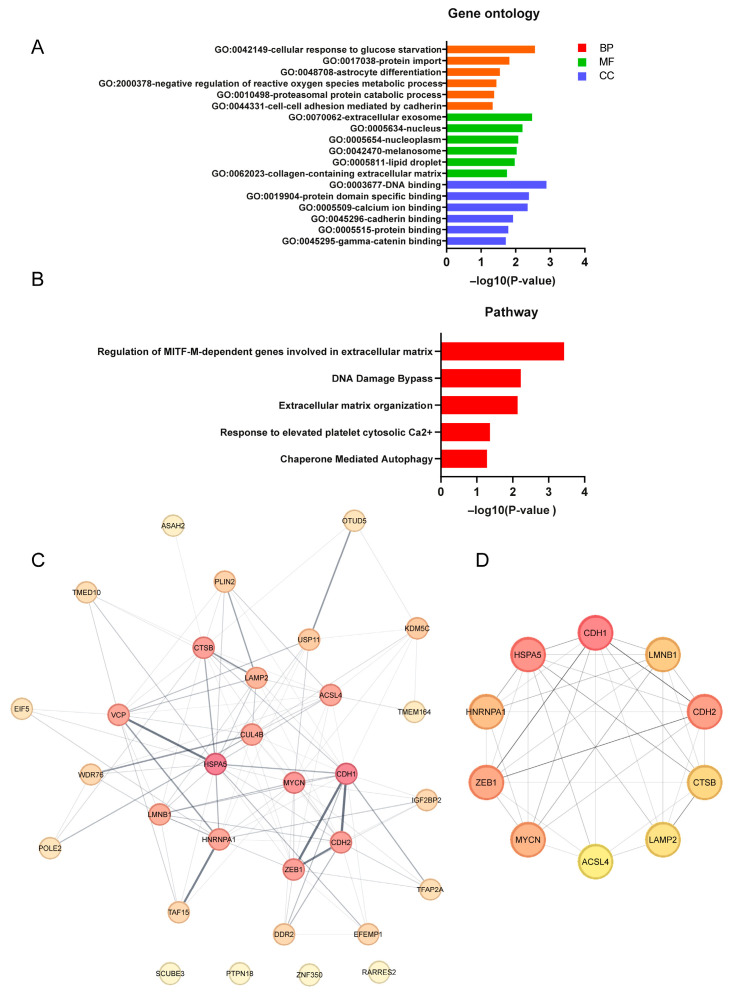
Functional enrichment analysis and PPI analysis of DEFRGs. (**A**) GO enrichment analysis of DEFRGs based on the top significantly enriched terms in each of the BP, CC and MF groups. (**B**) Pathway enrichment analysis of DEFRGs based on the top enriched pathways. The X-axis represent *p*-values, and the Y-axis indicates the GO/pathway terms. (**C**) Protein–protein interaction (PPI) network of the 31 candidate DEFRGs, in which the nodes represent proteins, and the edges represent the interaction of proteins. A thicker line indicates stronger data evidence, and genes/proteins of a darker color were distinguished using color shading from darker to lighter, according to the score (topological parameters). (**D**) Top 10 hub DEFRGs identified by the maximal clique centrality (MCC) algorithm using Cytoscape (cytoHubba plugin); the deeper the color, the higher the gene rank. GO, gene ontology; BP, biological process; CC, cellular component; MF, molecular function. For all analyses, *p* ≤ 0.05 was considered statistically significant.

To better comprehend the different pathways and biological functions of the 31 DEGs associated with FRGs in *FUS*-ALS, we performed GO annotation and KEGG enrichment analysis. GO enrichment analysis revealed that the biological process was primarily enriched in response to ROS, glucose starvation and RNA processing, while downregulated genes were mainly involved in protein import and protein catabolic processes. The cellular component comprised the nucleus/nucleoplasm (represented terms) and extracellular exosome (downregulated). Furthermore, the enriched molecular function contained DNA/RNA binding, while the depleted terms included protein domain binding and ubiquitin protein–ligase binding. The top six most significantly enriched terms in each of the categories were identified for the GO visualization bar graph (Figure 5A, Supplementary Figure S3). In addition, the pathway enrichment analysis revealed that DEFRGs were linked to extracellular matrix (ECM) and post-translational modifications (Figure 5B, Supplementary Figure S3), suggesting precise regulation of ferroptosis at multiple levels. The results obtained from GO/pathway enrichment analysis are shown in Supplementary Table S5.

### 3.6. Protein–Protein Interaction (PPI) Network Analysis of DEFRGs

To further explore the interactions of these identified 31 DEFRGs, we performed PPI network analysis. We obtained a PPI network consisting of 31 nodes and 110 edges, where network nodes represent proteins, and the edges represent protein–protein associations. The results showed that the 31 DEFRG decoded proteins were closely interconnected in the network (PPI enrichment *p*-value = 1.74 × 10^−4^) (Figure 5C), and these ferroptosis-related proteins were involved in biological functions such as the extracellular matrix and membrane bound organelle, suggesting that the mechanism related to ferroptosis may play an important role in the pathogenesis of *FUS*-ALS. Additionally, we have also identified several hub genes that have a high degree in the PPI network and which are ranked from high to low as follows, *CDH1*, *HSPA5*, *CDH2*, *ZEB1*, *MYCN*, *HNRNPA1*, *LMNB1*, *CTSB*, *LAMP2* and *ACSL4* (Figure 5D), highlighting that these genes played an important role in the core network. The interactions were visualized using Cytoscape software, and the PPI analysis results as well as scores of the DEFRGs (MCC algorithm) are listed in Supplementary Table S6.

## 4. Discussion

Although multiple mechanisms have been implicated in the pathogenesis of ALS, including glutamate excitotoxicity, mitochondrial dysfunction, oxidative stress, immune dysregulation and impaired axonal transport [44], underlying pathomechanisms causing MN degeneration and cell death pathways are poorly understood. Significant advancements have been made in microarray/RNA-sequencing analysis to identify gene expression profiles in different cells/tissues and contexts, thus helping to reveal important biological pathways under different conditions [28,45,46]. However, there have been few transcriptomic studies focused specifically on ferroptosis-associated genes and pathways in ALS.

For doing so, we compared our own and different available datasets to increase the number of biological replicates and generalizability. With that, we systematically explored the role of the ferroptosis-related gene signature in *FUS*-ALS. Briefly, we collected five comparable RNA-seq datasets from the GEO public database and performed integrated bioinformatic analysis to specifically look for mRNAs associated with ferroptosis and their related pathways that are differentially expressed in MNs from iPSC-derived *FUS* mutant-ALS patients and WT controls. A total of 672 common DEGs that are combined in five RNA-seq datasets were identified between *FUS* mutant and WT control MNs. The enrichment analysis revealed that DEGs were primarily misregulated in gene expression (transcription), organelle homeostasis, ER-protein folding and cell adhesion pathways. The accumulation of ROS is an important mechanism that can be the cause or consequence of mitochondrial dysfunction of MNs and can promote damage to the cell/organelle membrane, which leads to ferroptosis and other programmed cell deaths, such as apoptosis. Indeed, in GSEA analysis of RNA-seq data, we found that key genes involved in DNA repair, *p53* signaling, apoptosis, antioxidant activity, cellular response to ROS and stress-activated protein kinase signaling cascade were mostly enriched with higher enrichment scores, whereas gene sets related to mitochondrial function, cellular metabolism and ubiquitin/proteasome-mediated protein catabolic process were downregulated in *FUS* mutant MNs. These results suggest that increased cellular stress, caused by an imbalance between ROS and antioxidant defense systems, may induce apoptosis, and that played an important role in the pathogenesis of *FUS*-ALS.

Several studies have reported that *FUS* plays a crucial role in various cellular processes such as proteostasis, DNA repair (nuclear and mitochondrial), mitochondrial functions and RNA splicing or RNA metabolic processes [47,48,49]. Abnormal expression of *FUS* has been reported to exacerbate the accumulation of DNA damage, and *p53* is involved in activating DNA repair pathways. However, in case of prolonged DNA damage that is beyond repair, the *p53* promotes neuronal apoptosis by increasing the transcription of pro-apoptotic genes [47,49,50,51], suggesting a crucial role of *FUS* in the DNA repair-induced apoptosis mechanism in ALS pathology. Besides that, in a GSEA analysis, the degree of ferroptosis and iron ion homeostasis/response to iron of *FUS* MNs was lower, indicating that the genes related to ferroptosis were transcribed at lower levels compared to a WT control group. This downregulation of ferroptosis genes (a gene set) in the gene expression dataset suggests a decrease in the activity of ferroptosis (or less prevalent) in the *FUS* MNs, and this depletion could be due to multiple factors, including altered cellular metabolism or signaling pathways, various types of external stimuli, increasing antioxidant defenses and reducing iron availability, and even from changes in gene expression regulation that are altered in response to the *FUS* mutation, which was clearly evident from our GO/GSEA enrichment analysis of DEGs.

To further investigate a putative transcriptional activation of ferroptosis, we intersected the above-mentioned DEGs with FRGs collected from FerrDb and GeneCards. By this, we identified 31 potential DEGs (16 upregulated and 15 downregulated) related to ferroptosis (DEFRGs) between *FUS* mutant and WT controls. To understand the role of these DEFRGs in *FUS*-ALS, we further carried out GO/pathway enrichment and PPI analysis. The results of this analysis showed that the regulation of multiple biological functions and pathways, including increased ROS levels, regulation of gene expression (RNA polymerase II-specific) and stress response, were altered, which was in accordance with previous studies on ALS [52,53,54]. Taken together, the potential biological functions of DEFRGs in *FUS*-ALS involved the regulation of various signaling pathways and processes. The identified pathways might be triggered by upstream pathways, rather than being the primary pathway affected (e.g., ferroptosis) activated by a unique stimulus or response to a specific stress signal. Notably, the GSEA analysis of gene expression data showed that the ferroptosis pathway, including response to cellular iron/iron homeostasis, was downregulated in *FUS* MNs. It should be noted, DEFRGs also pointed towards a downregulation of ferroptosis (upregulated suppressor of the ferroptosis; downregulated driver of the ferroptosis). We assume that the collective effect of misregulated suppressors and drivers of ferroptosis may induce the decreased expression of ferroptosis at an early time point, while this might be different at later time points, which needs future investigations.

While a considerable amount of work has focused upon the metabolic adaptations that are found in *FUS* MNs and which enable specific adaptations, including apoptosis, metabolites, iron homeostasis and the need for protein homeostasis, the precise vulnerabilities of neuronal populations arising from these adaptations or alterations under physiological conditions to cell death remain poorly understood. In this context, through our gene expression studies of *FUS*-ALS datasets, we provided preliminary clues about the underlying cell death pathways to uncover the sensitivities of MNs to ferroptosis. On one hand, our enrichment analysis has revealed that DEGs in *FUS* MNs were primarily enriched in apoptosis or apoptosis-mediated functions. On the other hand, ferroptosis (or DEFRGs) showed reduced expression levels without significant enrichment of classical ferroptosis markers (e.g., *GPX4*) or ferroptosis-related processes, which suggest that *FUS* MNs are less prone to ferroptosis, at least at the time point of analysis. Despite the differences between the two cell death pathways, accumulating evidence suggests that ferroptosis and apoptosis can be induced by similar stress signals (e.g., ER stress or oxidative stress) and shared common regulators, such as *p53*. They may occur in the same damaged cells either sequentially or simultaneously and can even through their combined actions induce cell death [55,56]. As an essential gene, *p53* is required not only for transcription of the pro-apoptotic genes to various stress signals but also for the suppression of the anti-ferroptosis protein solute carrier family 7 member 11 (*SLC7A11*, a subunit of the cysteine–glutamate antiporter), which plays key role in cystine uptake and GSH metabolism [57]. Fitting to this, in GSEA analysis we identified upregulation of *p53*-signaling pathway and also found that the *SLC7A11* gene was downregulated in the ferroptosis gene set. Regulation of ferroptosis by *p53* is context-dependent (e.g., gene mutation and cell-type), can promote the presence of ferroptosis by inhibiting *SLC7A11* transcription, and can also reduce cell sensitivity to ferroptosis by acting on transcription-independent mechanisms [58]. A recent study showed that in cancer cells *p53* binds to and sequesters pro-ferroptotic enzyme dipeptidyl-peptidase-4 (*DPP4*) within the nucleus, forming an inactive complex, thus preventing its interaction with *NOX1* (NADPH oxidase 1) and decreasing lipid peroxidation/ferroptosis [59]. In other contexts, *p53* activation can lead to the expression of its downstream genes involving the cell cycle inhibitor *CDKN1A*/p21 pathway, which in turn increases intracellular *GSH*/*GPX4* levels to prevent lipid peroxidation and reduce ferroptosis sensitivity [60]. Further, in tumor cells *p53* also regulates ferroptosis sensitivity by upregulating the expression of a polyamine metabolism-related enzyme, spermidine/spermine N-acetyltransferase 1 (*SAT1*) [61]. However, the exact underlying mechanism of *p53* mediating reduced ferroptosis sensitivity in the above pathways is unknown and needs to be further elucidated. Further, in our differential enrichment analysis, gene expression and the stress response pathways were found in DEFRGs, while DEGs associated with the DNA repair/*p53* and with ubiquitin proteasomal response were connected to response to apoptosis and are consistent with the role of these specific pathways in each respective death program [16]. Although, the cellular consequences of ferroptosis regulation by *p53* could be complex, cell type-specific, bidirectional (positive or negative regulation), and context-dependent with distinct mechanisms, *p53* was proposed to play a critical regulatory role in the crosstalk between ferroptosis and apoptosis [56,58]. Thus, *p53* might be involved in activating DNA repair pathways (e.g., upregulation of DNA damage response genes or during extensive DNA damage) and may either trigger ferroptosis to help remove dead/damaged MNs or reduce the sensitivity of MNs to ferroptosis and promote normal cell survival, especially during the mild stress or injury. Our study’s main focus is to systematically investigate cell death activation pathways as well as cell adaptability in *FUS* mutant neurons to resist early forms of stress before MN loss is more obvious. Moreover, our own *FUS*-ALS datasets are from early time points where cells are viable before any visible signs or symptoms of cell damage occur [43,47,62]. Similarly, no early phase of cell stress or death has been observed in other *FUS*-ALS datasets reported in this study (Table 1), implying early time points are indeed crucial for capturing dynamic changes in signaling/survival cascades and gene expression events that forecast a cell’s fate [39,40,41,42]. Additional studies are required to explore how the compensatory mechanisms at harvest time relate to the long-term progression of the disease, where initial adaptations might eventually fail to counteract the damage.

While our results may provide new insights into an early transcriptomic regulation of FRGs, identifying potential pathways by using the data generated by different laboratories, they possess certain limitations and warrant further consideration. Firstly, this study relied mainly on the GEO database, including analysis of previously published datasets. Thus, the selection of datasets, lack of relevant clinical data/severity of the patients and batch-to-batch variation may differ from the interpretations of previous experiments, most likely due to potential biases caused by the small sample size, and more datasets are needed to confirm our findings. Secondly, the FRGs are sourced from the manually updated website FerrDb, and more relevant genes remain to be explored. Finally, our results are based on bioinformatic analysis, without experimental validation of DEFRGs. Therefore, future experimental studies are required to verify the reliability and significance of the DEFRG results to explore the complex regulatory network of ferroptosis underlying *FUS*-ALS pathogenesis.

## 5. Conclusions

In the present study, we performed a comprehensive bioinformatic analysis of currently available RNAseq datasets of *FUS*-ALS human MN datasets. By this, we identified 31 ferroptosis-related DEGs and identified their participating gene functions and pathways. We also detected key hub genes that were closely associated with ferroptosis and were mainly involved in signal transduction pathways and cell–cell adhesion functions. However, results point more towards ferroptosis being not the main cell death pathway activated—at least on transcriptional level—at the time point investigated. This does fit to the recent failure of the CardinALS trial, which is a phase 2 study on a lipoxygenase inhibitor in ALS (NCT05349721). In contrast, DEGs and GSEA enrichment analysis of gene expression data in *FUS* MNs highlighted the importance of the apoptosis pathway, which would also fit previous reports of increased apoptosis rates in *FUS*-ALS MNs [62]. Further studies—including longitudinal ones—are needed to finally unravel the sequence of cell death forms mainly contributing to MN death in (*FUS*-) ALS. Finally, future studies are warranted to investigate cell death pathways on a protein level.

## Figures and Tables

**Table 2:** The 11 overlapping genes (Up/Down) from the FerrDb and their role in ferroptosis.

Type	Genes
Driver	*CDH1* (Down), *DDR2* (Down), *ACSL4* (Down), *USP11* (Down), *ZEB1* (Up), and *MYCN* (Up)
Suppressor	*TFAP2A* (Up), *RARRES2* (Up), *PTPN18* (Up), *LAMP2* (Down), and *VCP* (Down)
Marker	None

## Data Availability

The original contributions presented in this study are included in the article/Supplementary Materials. Further inquiries can be directed to the corresponding author.

## Supplementary Materials

The following supporting information can be downloaded at: https://www.mdpi.com/article/10.3390/cells14181417/s1, Figure S1. Gene set enrichment analysis (GSEA) for differentially enriched terms and pathways (GO/KEGG) between FUS mutant and WT MNs (Upregulation). Figure S2. Gene set enrichment analysis (GSEA) of the GO/KEGG enriched terms between FUS and WT MNs (Downregulation). Figure S3. Functional enrichment analysis of DEFRGs. Table S1. Combined differential gene expression results of FUS mutant versus WT MNs. Table S2. Functional enrichment analysis (GO/KEGG) of DEGs between FUS mutant and WT MNs. Table S3. Gene set enrichment (GSEA) analysis of FUS mutant and WT MNs. Table S4. The list of FRGs and DEFRGs. Table S5. Functional annotation of DEFRGs using GO terms and pathway. Table S6. Protein-protein interaction network (PPI) analysis of DEFRGs.
