# Supplementary material for: Transcriptomic Alteration in FUS-ALS Points Towards Apoptosis-Rather than Ferroptosis-Related Cell Death Pathway
Source: Cells. 2025 Sep 10;14(18):1417. doi: 10.3390/cells14181417 (PMC12468769; doi:10.3390/cells14181417)
Supplement: Supplementary file 1 [file cells-14-01417-s001.zip › cells-3784481-supplementary-english.pdf]

Figure S1

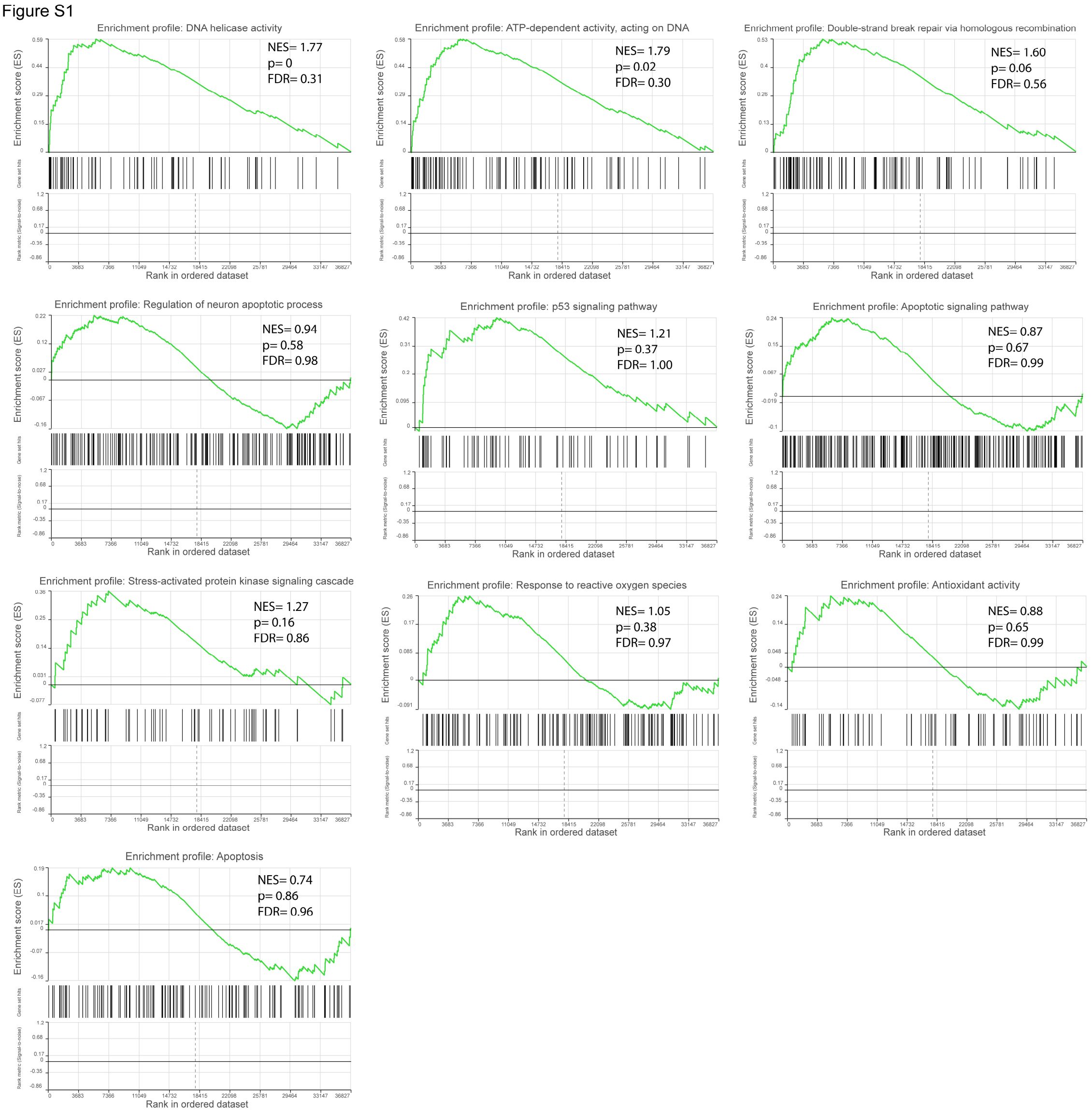

Figure S1. GSEA analysis for differentially enriched terms and pathways (GO/KEGG) between FUS versus WT MNs. The significantly enriched gene sets that positively correlated with the DEGs are based on normalized enrichment score (NES) values. GSEA plots were generated using normalized counts from the DESeq2 output ranked list in FUS versus WT (*p*-value ≤ 0.05). NES value represents the enrichment score after normalization. The higher the NES value, the more genes were enriched in the pathway. GSEA, gene set enrichment analysis; GO, gene ontology; KEGG, Kyoto encyclopedia of genes and genomes. Detailed information of the GSEA analysis can be found in Supplementary Table S3.

Figure S2

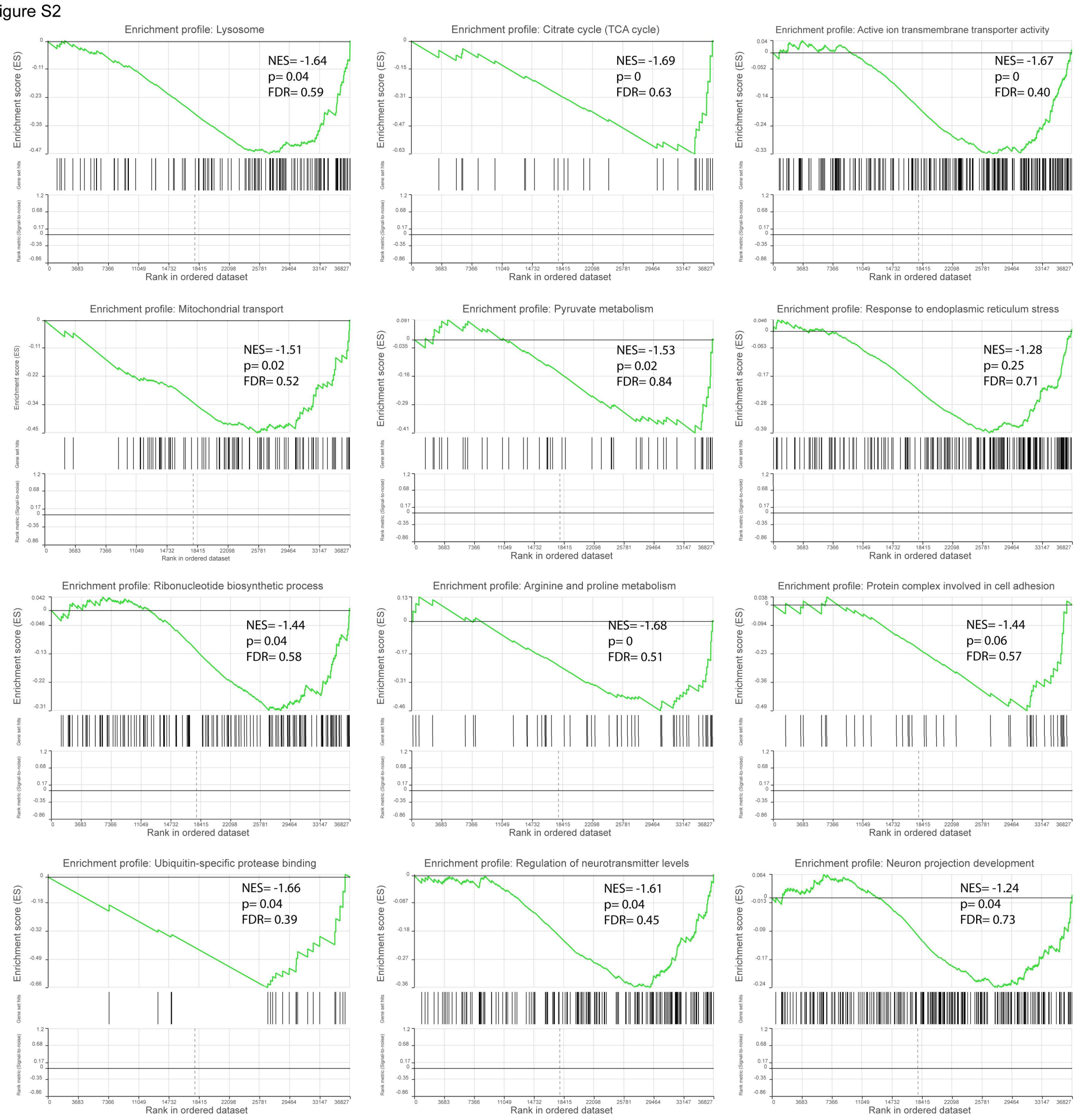

Figure S2. The significantly enriched gene sets that negatively correlated with the DEGs are based on NES value. GSEA plots were generated using normalized counts from the DESeq2 output ranked list in FUS versus WT (*p*-value ≤ 0.05). NES value represents the enrichment score after normalization. The higher the NES value, the more genes were enriched in the pathway. GSEA, gene set enrichment analysis; GO, gene ontology; KEGG, Kyoto encyclopedia of genes and genomes. Detailed information of the GSEA analysis can be found in Supplementary Table S3.

Figure S3

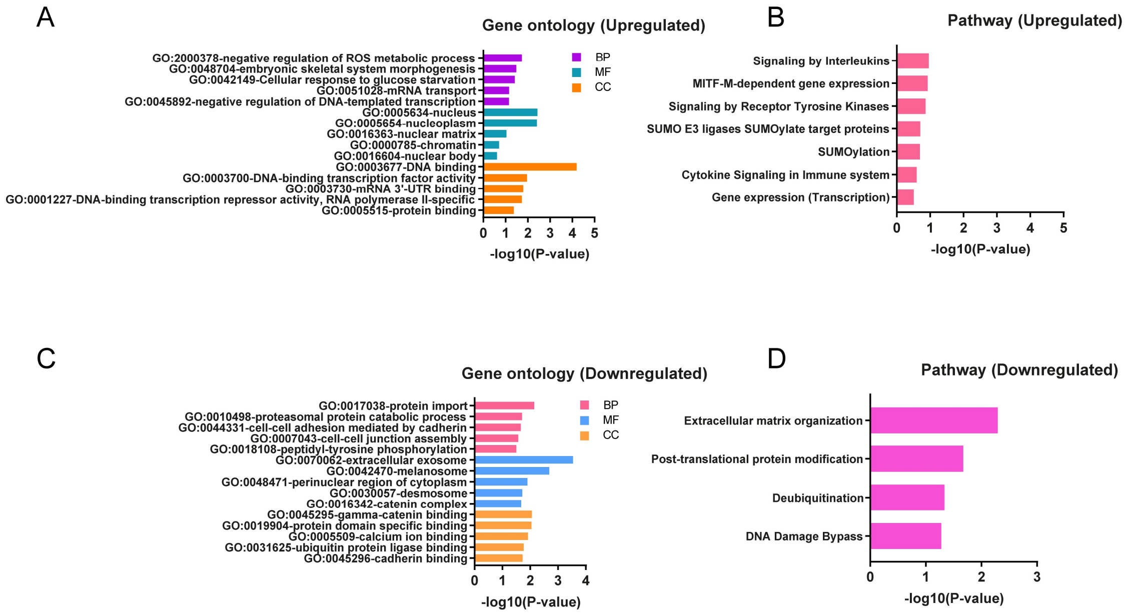

Figure S3. Functional enrichment analysis of DEFRGs. (A) Bar plot of gene ontology (GO) enrichment analysis of upregulated DEFRGs. (B) Pathway enrichment analysis of upregulated DEFRGs. (C) Bar plot of gene ontology (GO) enrichment analysis of downregulated DEFRGs. (D) Pathway enrichment analysis of downregulated DEFRGs. GO enrichment analysis was based on the top most significant enriched terms in each of the biological process (BP), cellular component (CC), and molecular function (MF) entries ($P \leq 0.05$).
